# Tumor-derived extracellular vesicles regulate macrophage polarization: role and therapeutic perspectives

**DOI:** 10.3389/fimmu.2024.1346587

**Published:** 2024-04-16

**Authors:** Lijuan Wang, Weihua Wang, Die Hu, Yan Liang, Zhanyu Liu, Tianyu Zhong, Xiaoling Wang

**Affiliations:** ^1^ The First School of Clinical Medicine, Gannan Medical University, Ganzhou, China; ^2^ Laboratory Medicine, First Affiliated Hospital of Gannan Medical University, Ganzhou, China

**Keywords:** extracellular vesicles, macrophages, M1, M2, colorectal cancer, glioblastoma

## Abstract

Extracellular vesicles (EVs) are important cell-to-cell communication mediators. This paper focuses on the regulatory role of tumor-derived EVs on macrophages. It aims to investigate the causes of tumor progression and therapeutic directions. Tumor-derived EVs can cause macrophages to shift to M1 or M2 phenotypes. This indicates they can alter the M1/M2 cell ratio and have pro-tumor and anti-inflammatory effects. This paper discusses several key points: first, the factors that stimulate macrophage polarization and the cytokines released as a result; second, an overview of EVs and the methods used to isolate them; third, how EVs from various cancer cell sources, such as hepatocellular carcinoma, colorectal carcinoma, lung carcinoma, breast carcinoma, and glioblastoma cell sources carcinoma, promote tumor development by inducing M2 polarization in macrophages; and fourth, how EVs from breast carcinoma, pancreatic carcinoma, lungs carcinoma, and glioblastoma cell sources carcinoma also contribute to tumor development by promoting M2 polarization in macrophages. Modified or sourced EVs from breast, pancreatic, and colorectal cancer can repolarize M2 to M1 macrophages. This exhibits anti-tumor activities and offers novel approaches for tumor treatment. Therefore, we discovered that macrophage polarization to either M1 or M2 phenotypes can regulate tumor development. This is based on the description of altering macrophage phenotypes by vesicle contents.

## 1 Introduction

Most cells in the body can secrete extracellular vesicles (EVs), which are small lipid bilayer vesicles with a diameter of 30–2000 nm (1). Since most EV separation techniques are unable to concentrate on EVs produced by different mechanisms, the updated version of the MISEV guidelines in 2023 discourages the use of biologically-generated terms, such as exosomes, microvesicles, etc. The International Society for Extracellular Vesicles(ISEV) recommends use of the generic term ‘EV’ and operational extensions of this term. ISEV continues to encourage the use of these terms for the variety of EV subtypes that are separated based on size, density, cellular origin, and other characteristics. Generally, small extracellular vesicles(sEVs) refer to EVs <200 nm in diameter, and large extracellular vesicles(LEVs) refer to EVs >200 nm in diameter. EV mimetics produced by inducing cell rupture in the laboratory are named artificial cell-derived vesicles (ACDVs), and EV mimetics synthesized from molecular components are named synthetic vesicles(SVs) (2). This review will be named using sEVs or LEVs according to the extracellular vesicle size of the original authors, as well as ACDVs or SVs depending on how they were synthesized. EVs will be used uniformly for cases where the size and production methods of vesicles are not specified in the original text, or when some concepts are being described. EVs contain genetic materials like proteins and nucleic acids, playing various roles in disease processes. These roles involve causing changes in recipient cells, communicating between cells, transferring proteins and nucleic acids, impacting inflammation and immune regulation, and influencing angiogenesis and coagulation (3, 4).

EVs are crucial for studying disease pathogenesis, prognosis, and diagnostic due to their ability to carry biologically active cargo. Hematopoietic cells, including B cells, platelets, macrophages, and dendritic cells, produce sEVs (5). Additionally, non-hematopoietic cells, such as tumor cells, Schwann cells, astrocytes, etc., produce it (6–8). Several studies have demonstrated that sEVs alter the macrophage phenotype.

The active molecules that EVs contain determine the different phenotypes of macrophages. The complex junction of various cells that determine the tumor microenvironment (TME)—also referred to as the tumor stroma—is where cancer cells interact with surrounding cells. Immune cells, stromal cells, blood vessels, and extracellular matrix are characteristics of TMEs (9). Macrophages are found in all tissues and are essential components of the TME. As innate immune cells, macrophages can polarize in different directions based on their surrounding environment (10). Additionally, macrophages are found in all tissues. As a result, they are essential cellular components of TME (11). Macrophages play a role in the development and progression of tumors by promoting angiogenesis (12), facilitating tumor cell migration and invasion (13), and enhancing tumor drug resistance (14). Macrophages can undergo polarization and differentiate into M1 and M2 macrophages in response to alterations in the TME (15). When M1 macrophages are activated, they release pro-inflammatory factors like tumor necrosis factor-alpha (TNF-α), interleukin-6 (IL-6), and IL-12. Macrophage polarization is activated to the M1 phenotype through lipopolysaccharide (LPS) and interferon-gamma (16, 17). These factors also have anti-tumorigenic and pro-inflammatory properties. IL-4 and IL-13 promote M2 macrophages to polarize to the M2 phenotype, which releases several anti-inflammatory factors to reduce the inflammatory response (18, 19). M2 macrophages have anti-inflammatory and tumor-promoting effects. However, the relative roles of M1 and M2 macrophages significantly affect tumor development and therapy in the TME (20, 21). EVs produced from tumor cells are believed to facilitate communication between tumor cells and immune cells (22). Changes in immune cell phenotype in the TME can directly impact tumor progression (23). This paper focuses on how EVs from tumor cells influence macrophage polarization, affecting tumor progression.

## 2 Macrophage polarization

In addition to malignant cells, TME contains many normal cells, such as blood vessels, fibroblasts, and immune cells. Macrophages are significant immune cells in this milieu. In the TME, these malignant cells interact with the active factors secreted by normal cells (24). The primary characteristic of the TME is immunosuppression, which changes as the tumor progresses. For example, certain primary tumors release multiple molecules that contribute to cancer-promoting TME, facilitating tumor cell colonization and dissemination to distant organs (25). However, it is challenging to target tumors precisely because the TME has many commonly expressed cells that suppress the immune response. These are the reasons why TME-induced tumor treatment can be challenging. Although macrophages are the most abundant critical non-tumor immune cells in the TME, research into the mechanism of tumor-macrophage interaction may pave the way for novel approaches to tumor therapy (26).

Tumor-associated macrophages (TAMs) comprise 30% and 50% of the total tumor mass and are the most prevalent and significant non-tumorigenic cells in the TME (27). How macrophages polarize towards anti-inflammatory pro-tumorigenic M2 or pro-inflammatory anti-tumorigenic M1 determines whether the TME has anti-tumor or pro-tumorigenic effects. Additionally, the stimulation of TME influences the polarization of these macrophages. TAMs are essentially groups of macrophages with distinct phenotypes. When TAMs take on an M2 phenotype, they promote angiogenesis, immunosuppression, and invasive metastasis, contributing to tumor development (28). Strategies for treating TAMs-associated tumor immunotherapy include polarizing M2-like TAMs to M1 anti-tumor phenotype, reducing the survival of M2 phenotype TAMs, and inhibiting the recruitment of infiltrating macrophages (29, 30). The first of these approaches is the most straightforward.

**Figure 1:**
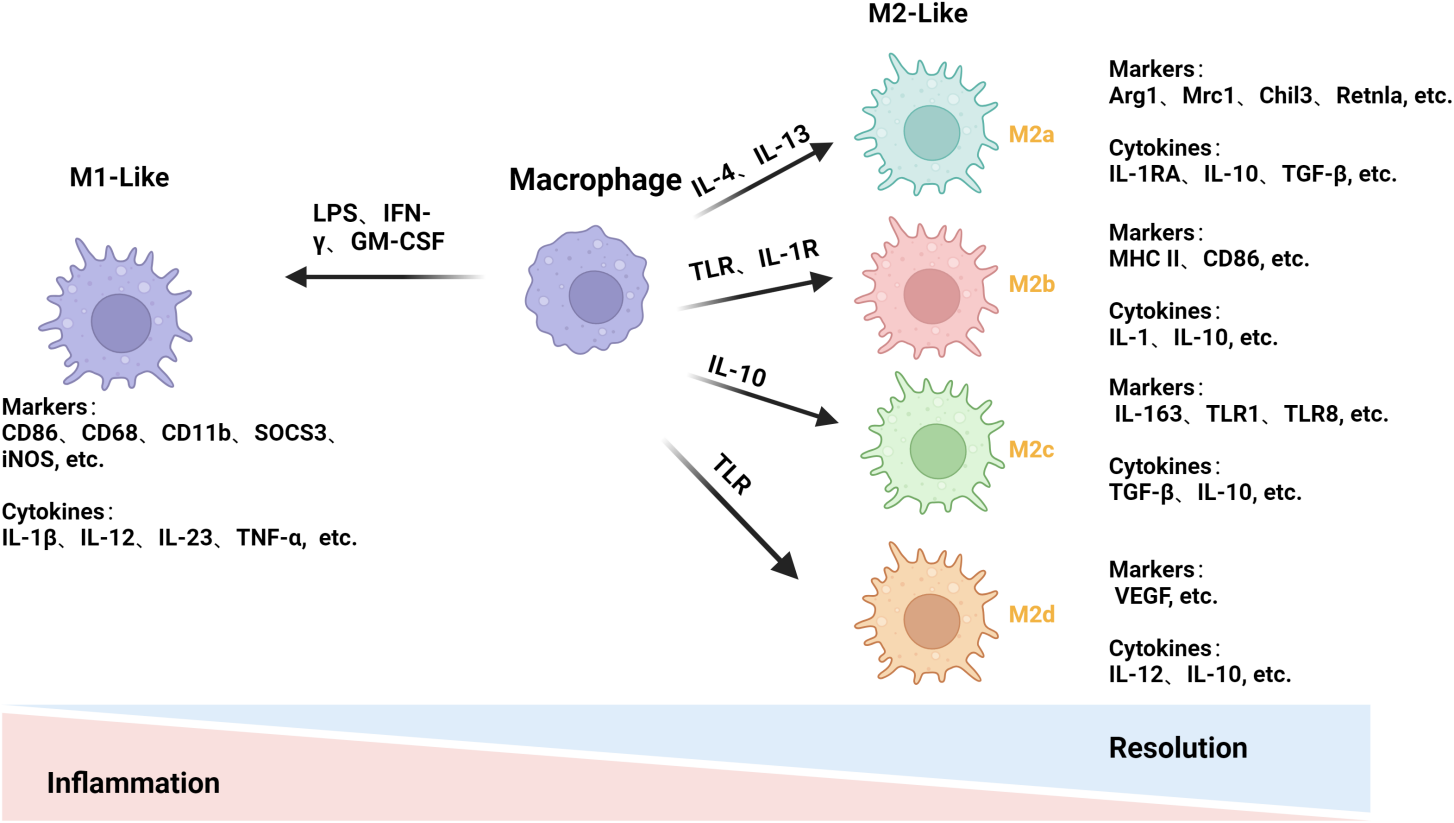
Mechanisms of macrophage polarization. LPS and others stimulate macrophage polarization to the M1 phenotype to release pro-inflammatory factors for pro-inflammatory and anti-tumor effects. IL-4 and others stimulate macrophages to differentiate into the M2 phenotype and release anti-inflammatory factors to achieve anti-inflammatory and pro-tumorigenic effects. The M2 phenotype is subdivided into four subtypes: M2a, M2b, M2c, and M2d. (This figure was created with Biorender.com.)

Macrophages exhibit different phenotypes that are broadly classified into two types: classically activated M1-like macrophages and alternatively activated M2-like macrophages, depending on the TME (31, 32). In the early stages of the disease, macrophages often adopt a pro-inflammatory M1 phenotype; however, as the disease progresses, they adopt an anti-inflammatory M2 phenotype to promote tissue repair, regeneration, and fibrosis. Disease development is closely associated with the transition between the M1 and M2 phenotypes, and the two phenotypes each have unique roles that interact to regulate the development of the disease (**Figure 1**) (33).

### 2.1 M1-like macrophages

M1 macrophages can present antigens and have pro-inflammatory effects (34). The M1 phenotype is induced by stimuli such as LPS, IFN-γ, and granulocyte monocyte colony-stimulating factor. This allows the M1 phenotype to play a significant role in the early stages of inflammation. Pro-inflammatory cytokines, such as IL-1β, IL-12, IL-23, and TNF-α, are released, further promoting inflammatory and cytotoxic responses (35, 36). To track changes in macrophage M1-like polarization, we often look for changes in relevant markers, including CD86, CD68, CD11b, suppressor of cytokine signaling 3, inducible nitric oxide synthase (iNOS), etc. (37, 38).

### 2.2 M2-like macrophages

M2 macrophages can be subdivided into M2a, M2b, M2c, and M2d. M2 macrophages have a low antigen-presenting ability and mostly release anti-inflammatory factors (39). IL-4 or IL-13 can effectively counteract inflammatory damage and promote tissue healing by stimulating macrophages to differentiate into M2a cells and release anti-inflammatory cytokines such as IL-1RA, IL-10, and transforming growth factor-beta (TGF-β) (40, 41) via the co-receptor IL-4Rα. Arg1, Mrc1, Chil3, and Retnla are their primary markers of detection (42). When TLR or IL-1R agonists are exposed to the body, they produce M2b macrophages that release cytokines, IL-10, IL-1, etc., to exert immunomodulatory effects. Major histocompatibility complex II (MHC II), CD86, etc., are their primary monitoring markers (41, 43). IL-10 helps macrophages become M2c. IL-10 helps macrophages become M2c, which has anti-inflammatory and anti-phagocytic effects, which stimulates macrophages to induce an M2c phenotype and produce cytokines like TGF-β and IL-10. This process also induces arginase-1 (Arg1) expression. CD163, TLR1, TLR8, etc., are the primary indicators for its detection (44, 45). TLR agonist-induced macrophages primarily release cytokines such as IL-10 and IL-12, which promote tumor progression and angiogenesis and are the primary source of M2d macrophages (46). Vascular endothelial growth factor and other primary monitoring markers (47). Different types of M2 macrophages have different roles, so it is crucial to study them separately.

## 3 Extracellular vesicles

### 3.1 Overview of extracellular vesicles

Sheep reticulocytes are the source of the EVs that Pan (48) and Johnstone (49) first discovered and dubbed “exosomes”. EVs are released by all types of cells, including blood, immune, cancer, and stem cells. Blood, urine, breast milk, ascites, amniotic fluid, saliva and cerebrospinal fluid are among the bodily fluids into which the stem cells created by these cells can be released (50). Proteins and nucleic acids (miRNA, mRNA, and lncRNA) are abundant in content found in EVs. Due to their genetic material carrying capacity, EVs are crucial in various diseases (51).

### 3.2 Techniques for isolation of extracellular vesicles

There is disagreement on the best extraction technique for EVs, although they are extracted using various techniques. Differential ultracentrifugation(dUC), size exclusion, immuno-capture, and precipitation are often employed techniques (52). When selecting an isolation approach, we must have a thorough grasp of the downstream needs of our research because each method has pros and cons of its own (53, 54). Our method for isolating EVs is selected based on our research needs for harvest rate and specificity. Due to its high harvest rate and low cost, dUC is the most commonly used method for isolating EVs. However, it has drawbacks, like being time-consuming and having limited specificity (55). Understanding that specificity and harvest rate have an inverse relationship is essential. As a result, no single technique can accomplish a high harvest rate and high specificity. The latest MISEV guidelines suggest that large volumes of source materials may require concentration before EVs can be separated from other extracellular particles (EPs), a move that may make some separation methods more efficient. Notably, the guidelines specifically note that precipitation methods may not be able to separate different types of EPs; for example, the Exosome Separation Kit relies heavily on polymer precipitation to separate EVs, and in fact these kits do not rigorously separate EVs, let alone other subtypes of EVs (2). The principles, advantages and disadvantages of various commonly used separation methods are mainly summarized below: (i) dUC is to apply increasing relative centrifugal forces to the EV‐containing fluid by centrifuging multiple times to separate vesicles and other EPs and by controlling the magnitude of centrifugal force and centrifugation time to distinguish between vesicles of different diameters. Typically we use low speed centrifugation to remove cellular debris and large vesicles (typically 10,000 to 2,000g) and ultra-high speed centrifugation for smaller vesicles (typically 100,000 to 200,000g). This method is the most commonly used and inexpensive, but it is very time-consuming, often requiring 60-120 min to extract the different subtypes of EVs needed, and has low specificity (53, 56). (ii) Density gradient centrifugation is a more rigorous form of ultra-centrifugation, where vesicles of different densities settle at different rates on a gradient. EV‐containing materials can be loaded beneath a gradient or onto the top of a gradient or cushion and then ultracentrifuged. After the EVs preparation is loaded below the gradient for a sufficiently long period of ultracentrifugation, the particles will eventually reach a density fraction corresponding to their buoyant density. Since smaller EVs run slower than larger ones, this method can be used to separate sEVs from LEVs. Density gradient method is also one of the more commonly used methods, which has high specificity for the extraction of EVs. However, the method has low yield and is very time-consuming, even requiring 16-48h to complete the whole experiment (57). (iii) Precipitation involves the use of chemicals such as polyethylene glycol to reduce the solubility of the EVs, thereby causing them to precipitate, followed by low-speed centrifugation to obtain sEVs. This method, although rapid in extraction, is particularly susceptible to the introduction of new contaminants such as polyethylene glycol and makes it difficult to distinguish between EPs and EVs (2). (iv) Size exclusion uses a column with a defined pore size to separate EVs of different particle sizes. Driven by gravity, larger EVs do not enter the pore and are quickly eluted through the column at an early stage, whereas the opposite is true for smaller EVs, so that different sizes of EVs can be collected by changing the substrate. This method allows for the rapid separation of EVs, but has a limited lifetime of the column, is costly to extract and often carries contaminating proteins (58). (v) Immunocapture involves the separation of vesicles coated with magnetic beads containing the target protein. The method is rapid but costly to extract (59). The above methods can be used to isolate EVs and different subtypes of EVs, especially LEVs and sEVs, can be isolated by changing the experimental conditions such as centrifugation speed, time, column pore size, etc. For characterization of the extracted EVs, the most recent guidelines state that there is no universal molecular marker for the identification of a specific subtype of EVs. In the current literature, studies of EVs have focused on smaller diameters, and commonly used identification tools include nanoflowmetry to detect the diameter of extracted vesicles, protein blotting to detect marker proteins of EVs (e.g., positive markers CD63, CD9, Alix, and negative marker GM130) (60), and transmission electron microscopy to observe the microscopic morphology of the vesicles (61).

## 4 Extracellular vesicles induce M2 polarization in macrophages

The development of tumors depends heavily on the TME. Macrophages are immune cells linked to the advancement of tumors (62). These macrophages are thought to be essential for the evolution of tumors because EVs promote polarization in macrophages, which can then be engaged in tumor proliferation, invasion, angiogenesis, and tissue inflammation through pertinent downstream pathways (63, 64). Macrophages activate tumor-associated fibroblasts, pro-angiogenic factors, and other components of the TME (65, 66).

Tumor-derived EVs can be absorbed by all immune cells and are essential in immunomodulation and TME (67). M1 and M2 macrophages can change in response to EVs, affecting the TME. According to studies, TAM is an M2 phenotype implicated in tumor angiogenesis, proliferation, and metastasis (68). Tumor-derived EVs have been proposed as a critical communication mediator between tumors and immune cells. Next, we will discuss how several tumor-derived EVs, enumerated in **Table 1**, contribute to macrophage M2 polarization and tumor growth.

**Table 1:** Role of tumor-derived extracellular vesicles in macrophage polarization and the role.

Macrophage phenotype	Cancer type derived EVs	EVs cargo	Major outcome	References
**M1**	Breast cancer	miR-33	miR-33-containing SVs could reprogram macrophages for anti-tumor effects	Moradi-Chaleshtori M, Bandehpour M et al.
		miR-33 and miR-130	Reprogram macrophages for anti-tumor effects	Moradi-Chaleshtori M, Bandehpour M et al.
		unrevealed	Repolarizing macrophages towards the M1 macrophages	Ghalavand M, Moradi-Chaleshtori M et al.
	Colorectal cancer	antisense oligonucleotides targeting STAT6	Silence the expression of STAT6 in macrophages to convert macrophages from M2 to M1	Kamerkar S, Leng C et al.
		unrevealed	M2 macrophages to shift to the M1 phenotype	Stary V, Wolf B et al.
	Pancreatic cancer	miR-155 and miR-125b-2	M2 macrophages shift to the M1 phenotype and exert anti-tumor invasion and metastasis effects	Su MJ, Aldawsari H et al.
**M2**	Breast cancer	circ_0001142	sEVs carrying circ_0001142 induce M2 polarization and interfere with autophagy levels	Lu C, Shi W et al.
		miR-138-5p	Inhibition of KDM6B expression by miR-138-5p induces M2 polarization	Xun J, Du L et al.
		miR-222	sEVs carrying miR-222 promote macrophage M2 polarization through activation of the PTEN/AKT pathway	Chen WX, Wang DD et al.
	Hepatocellular carcinoma	lncRNA PART1	EVs carrying lncRNA PART1 inhibit miR-372-3p to upregulate TLR4 expression to promote M2 polarization in macrophages	Zhou J, Che J et al.
		miR-452-5p and miR-200b-3p	Promote macrophage M2 polarization	Zongqiang H, Jiapeng C et al. and Xu Y, Luan G et al.
		miR-21-5p	Promote M2 polarization and hepatocellular carcinoma proliferation	Yu H, Pan J et al.
	Lung cancer	miR-19b-3p, miR-138-5p and miR-3153	microRNAs carried by sEVs of tumor cell origin that can induce M2 polarization in macrophages	Chen J, Zhang K et al. and Xun J, Du L et al. and Xu L, Wang L et al.
		miR-21	Promote macrophage M2 polarization and upregulate tumor growth rate	Jin J and G.
		unrevealed	sEVs promote lung cancer progression by regulating macrophage polarization through upregulation of miR-1290	Gu J, Yang S et al.
	Glioblastoma	circ_0012381	Irradiated GBM cells induced microglia M2 polarization mainly by secreting circ_0012381-containing sEVs	Zhang C, Zhou Y et al.
		miR-27a-3p	sEVs can induce M2 polarization in macrophages via miR-27a-3p	Zhao G, Yu H et al.
	Colorectal cancer	miR-934 and miR-203a-3p	Acts as a tumor marker and induces macrophage M2 polarization by downregulating PTEN expression	Zhao S, Mi Y et al. and Pei W, Wei K et al.
		ciRS-122	Reversing chemoresistance	Wang X, Zhang H et al.
		miR-106b-5p	Acts as a tumor marker and induces macrophage M2 polarization	Yang C, Dou R et al.
	Pancreatic cancer	miR-301a	Acts as a tumor marker and induces macrophage M2 polarization by downregulating PTEN expression	Wang X, Luo G et al.
		miR-155-5p	Induces macrophage M2 polarization	Wang S and Y.

### 4.1 Breast cancer-derived extracellular vesicles

Breast cancer is one of the most common malignant tumors in women globally, with a high rate of mortality and morbidity. Research has demonstrated that breast cancer cell-derived sEVs can induce M2 polarization in macrophages by the transfer of circ_0001142 and can also disrupt autophagy levels, promoting tumor proliferation, migration, invasion, and epithelial-mesenchymal transition (69). MiRNAs in tumor cells can regulate macrophage polarization using EVs vectors and circRNAs. The 3’-untranslated region of KDM6B was the target of increased production of miR-138-5p in EVs in breast cancer cell-derived EVs, which promoted M2 polarization by inhibiting the expression of KDM6B in macrophages. When Xun et al. transplanted mouse macrophages from EVs-treated breast cancer cells overexpressing miR-138-5p into mice for *in vivo* experiments, the experimental group displayed more lung metastases than those transplanted with mouse macrophages from control EVs (70). In sEVs derived from adriamycin-resistant breast cancers, Chen et al. found that miR-222 expression was significantly higher. They found that the levels of miR-222 were significantly higher in tumor tissues of patients resistant to chemotherapy compared to those sensitive to chemotherapy. This suggests that miR-222 could serve as a predictor of tumor progression. Resistant breast cancer-derived sEVs containing miR-222 promote macrophage M2 polarization and shift the role of macrophage against tumors from inhibition to promotion by activating the PTEN/AKT pathway. Additionally, they implanted a mixture of macrophages treated with adriamycin-resistant tumor cell sEVs subcutaneously in mice. They found that the subcutaneous tumors in the sensitive group were considerably smaller than those in the adriamycin-sensitive group (71).

### 4.2 Hepatocellular carcinoma-derived extracellular vesicles

HCC is the sixth most prevalent cancer globally and is third in terms of overall cancer mortality. Zhou et al. reported that EVs from liver cancer cells can be ingested by macrophages and cause them to upregulate TLR4 expression by inhibiting miR-372-3p. This is achieved by the EVs carrying PART1, a long-stranded noncoding RNA (lncRNA) that promotes M2 polarization in macrophages. Consequently, M2 macrophages significantly increased the tumorigenicity of HCC cells in nude mice, and lncRNA PART1 also promoted the proliferation, migration, invasion, and epithelial cell transformation (EMT) of tumors (72). MiR-452-5p and miR-200b-3p were found in high levels in EVs of HCC cells. They are linked to tumor progression, survival, and recurrence. Moreover, they promote the growth of hepatocellular carcinoma and can promote M2 polarization of macrophages. Following the injection of tumor cell-derived EV inhibitors or miRNA mimics into mice, the mice treated with EV mimics exhibited higher tumor volumes and more significant lung nodal metastases (73, 74). HCC cell-derived sEVs that express miR-21-5p aggregates in macrophages directly target RhoB to promote M2 polarization in macrophages and promote hepatocellular carcinoma proliferation (75).

### 4.3 Lung-cancer-derived extracellular vesicles

Lung cancer is among the world’s most common causes of cancer-related deaths, with its high aggressiveness and inferior prognosis. Regarding the pathogenesis of lung cancer, in addition to the microRNAs, such as miR-19b-3p, miR-138-5p, and miR-3153 that are carried by EVs originating from tumor cells and can trigger M2 polarization in macrophages and promote the development of lung cancer (70, 76, 77). Meanwhile, hypoxia can change the course of a tumor and is a common occurrence in the TME. Increasing research indicates that tumor cells may be stimulated to produce sEVs under hypoxic conditions to promote tumor progression. According to Jin et al., miR-21 expression was more abundant in hypoxic lung cancer-derived sEVs. These miR-21 were also found to be translocated to macrophages, where they bound to interferon regulatory factor 1 and inhibited its expression. This process can promote macrophage M2 polarization and upregulate tumor growth rate. In experiments with male nude mice, researchers injected a mix of H1299 cells and THP-1 cells under different conditions. Mice treated with sEVs from hypoxic H1299 cells showed higher levels of miR-21 expression and larger tumor volumes. Immunohistochemistry (IHC) analysis of the tumor tissues from mice in each group treated showed that the hypoxic sEVs-treated group had higher levels of CD163 expression in their tissues, suggesting a higher level of M2 macrophage infiltration (78). Gu et al. showed that hypoxic lung cancer cell-derived sEVs promote lung cancer by influencing macrophage polarization through increased miR-1290 levels. They measured tumor volume and weight in different groups using a mouse model and detected CD163 expression in the tissues using IHC. This showed that tumor tissues in mice treated with hypoxic cancer cells sEVs had increased in volume, weight, and CD163 expression (79).

### 4.4 Glioblastoma cell-derived extracellular vesicles

Although pilocytic astrocytomas, the least malignant of these, only have a 5–10 year survival rate, gliomas make up 80% of all primary tumors (80). Glioblastoma (GBM) is the most malignant type of glioma; it is highly aggressive and rapidly progressive and only gives patients a 15-month survival period after diagnosis (6.8%) (81). Following surgery, radiotherapy, and chemotherapy, patients with GBM eventually relapse, exhibiting a high degree of aggression and drug resistance. We suggest that immunosuppression may have a role in the poor response to GBM therapy (82). High levels of EVs released by GBM cells have been shown to serve as a diagnostic marker in the blood of patients with GBM (83). TAM cells originate from two primary sources: bone marrow-derived and microglia cells. In the GBM microenvironment, TAM cells are primarily derived from the latter. The crosstalk between GBM and microglia significantly influences tumor progression and therapeutic resistance. In a recent study by Zhang et al., microglia were found to absorb sEVs released by irradiated GBM cells, inducing them to undergo M2 polarization. This lowers the microglia’s ability to phagocytose microglia. The increased CCL2 expression in M2 microglia also notably supported the proliferation of irradiated GBM cells. Furthermore, the study observed an increase in circ_0012381 in sEVs derived from irradiated GBM cells, suggesting that these sEVs, containing circ_0012381, primarily induce M2 polarization in microglia. This offers a potential direction to improve the efficacy of radiotherapy. To confirm the findings of this experiment *in vivo*, they injected zebrafish embryos with a mixture of GBM cells and microglia and incubated them. Following that, they selected similarly sized fry to be exposed to radiation. At the end of the experiment, they used fluorescence microscopy to measure the tumor volume of the zebrafish. They found that the group of glioma tumor cells treated with the exosome inhibitor GW4869 ad notably reduced tumor weight and volume compared to the control group. This increased the efficacy of radiation therapy (84). Research has demonstrated that GBM cell-derived sEVs and GBM tissues have higher expression levels of miR-27a-3p. These microRNAs are transferred from sEVs to macrophages, inhibiting the zeste homolog 1 (EZH1) expression by targeting it. This process, mediated by miR-27a-3p, prompts M2 polarization in macrophages, which enhances proliferation, migration, and resistance to radiotherapy in GBM tumors. In addition to their contribution to tumor pathogenesis, they have the potential to serve as diagnostic markers for GBM (85).

### 4.5 Colorectal cancer-derived extracellular vesicles

CRC is the second most common cause of tumor-related deaths globally, with the third highest prevalence. Liver metastases from CRC account for a high number of deaths. In a study by Zhao et al., higher levels of miR-934 were found in the serum of CRC patients, with or without liver metastases, and in healthy individuals. This elevated expression of miR-934 may suggest the presence of liver metastases in CRC. Subsequently, they investigated the pathogenesis of this molecule in CRC cell-derived EVs. They discovered that via downregulating PTEN expression, miR-934 in EVs generated from CRC cells caused macrophage M2 polarization, CRC invasion, and metastasis (86). Pei et al. found that miR-203a-3p in sEVs from CRC cells act similarly to what was mentioned earlier by targeting PTEN to promote M2 polarization and assist in CRC hepatic metastasis. Additionally, they performed plasma analysis and discovered that CRC cell-derived sEVs containing miR-203a-3p might be used as a non-invasive fluid marker to identify liver metastasis (87). Therefore, we found that miRNAs in sEVs can strongly indicate diagnostic markers and trigger the onset of CRC, which could help with the subsequent CRC diagnosis and treatment. Of course, in addition to being a highly invasive and metastatic tumor in and of itself, CRC has a low survival rate and is a challenging case of chemoresistance. Aerobic fermentation produces ATP, which aids in rapid tumor growth and chemoresistance in malignant tumors. Wang et al. showed that tumor cell lines from oxaliplatin-resistant tumors can accelerate glycolysis and drug resistance by releasing sEVs containing ciRS-122, which upregulates ciRS-122 levels in sensitive cell lines through transcellular transfer. To separate the mice into experimental and control groups, they transplanted drug-resistant and sensitive tumor blocks into the right inguinal region of female nude mice. They transplanted SW480 tumor blocks of the same size into each inguinal region. They then injected oxaliplatin into the peritoneal cavity of each group of mice. They cut off the left side of the tumors to measure the volume, which was more significant than that of the control group. This study may provide directions for reversing chemoresistance in CRC (88). EMT increases the ability of cells to invade other cells and metastasize, which contributes to cancer development. According to Yang et al., there was an increase in the expression level of miR-106b-5p in the sEVs of EMT-CRC cells. This expression level is essential because it can target programmed cell death 4, activate the mammalian target of the rapamycin signaling pathway, and promote macrophage M2 polarization. Meanwhile, CRC cell migration, invasion, and metastasis mediated by EMT may be inhibited by activated M2 macrophages. Additionally, they looked at miR-106b expression in plasma sEVs and found that it was increased and correlated with CRC malignancy (89).

### 4.6 Pancreatic cancer-derived extracellular vesicles

Despite treatment, less than 5% of patients with pancreatic cancer survive for five years, making it one of the most dangerous cancers. The high rate of mortality and the advanced stage of the disease at the time of diagnosis are the primary causes of the high death rate among individuals with pancreatic cancer. Thus, research into the pathogenesis of pancreatic cancer and the development of new diagnostic markers are essential. An essential part of tumor metastasis is played by hypoxia and inflammatory cell infiltration. Wang et al. found that sEVs produced from pancreatic cancer cells could transfer miR-301a to macrophages by targeting ETS homologous factor. This promotes M2 polarization in macrophages by downregulating PTEN expression. They discovered miR-301a in serum sEVs and could be used as a diagnostic marker. They also proposed that miR-301a in sEVs may be exploited as a target for tumor immune escape (90). One of the main factors contributing to pancreatic cancer treatment challenges is immune evasion. Wang et al. discovered that EVs produced from pancreatic cancer cells could transfer miR-155-5p to macrophages through their targeting ETS homologous factor. This resulted in the activation of the Akt/NF-κB pathway, which enabled the macrophages to become M2 polarized and promoted tumor escape (91).

## 5 Extracellular vesicles induce M1 polarization in macrophages

M1 polarized macrophages have pro-inflammatory and anti-tumor properties (92). As nanocarriers, EVs can boost immune cell reactions against cancer. Thus, introducing EVs from donor cells into macrophages may cause M1 polarization in these cells, which has anti-tumor properties. Based on previous research, we discovered that proteins might be produced exogenously loaded onto EVs to drive them to particular target cell sites, in addition to the therapeutic benefits reported in naturally occurring EVs (28, 93). The pro-tumor phenotype M2 phenotype of macrophages is changed into an anti-tumor M1 phenotype by this EVs-based immunotherapy, which shows promise in slowing the growth of tumors (**Table 1**) (94).

### 5.1 Breast cancer-derived extracellular vesicles after artificial modification or treatment

Over the past 50 years, significant progress has been made in the diagnosis and treatment of breast cancer. Moradi-Chaleshtori et al. allegedly changed M2-polarized macrophages into M1 macrophages by electroporating sEVs from breast cancer 4T1 cell lines with miR-33 mimics during co-culturing with IL-4-induced M2-type macrophages. They concluded that macrophages could be reprogrammed for anti-tumor activities by introducing SVs expressing miR-33 (95). In a related study, they encapsulated miR-33 and miR-130 into sEVs to reprogram macrophages to produce more indicators of their M1 phenotype, which allowed them to exert anti-tumor activities. They injected mice with a mixture of 4T1 cells and macrophages from various treatment groups to observe how sEV-treated macrophages affected the development of breast cancer. The tumor volume was less, and patches of necrosis in the tumors of these mice were also visible for anti-tumor effects, as demonstrated by the eosin staining comparing the mice receiving miR-130 or miR-33-loaded SVs or both-treated macrophages to the control group (96). Finding more effective treatments is critical since triple-negative breast cancer makes up 10%–24% of all breast cancers in clinical practice and has a poor prognosis, significant drug resistance, and high metastasis. Ghalavand et al. treated the breast cancer 4T1 cell line with rapamycin and isolated sEVs to influence M2 macrophages produced by IL-4, repolarizing them towards M1 macrophages. This enhanced macrophage phagocytosis. These findings lift the immunosuppression of the tumor by promoting macrophage M1 polarization, which offers a fresh approach to clinical tumor therapy (97).

### 5.2 Colorectal cancer-derived extracellular vesicles after artificial modification or treatment

Reprogramming TAM cells to the M1 phenotype is one novel way to generate anti-tumor immunity for treating colorectal cancer. Numerous researchers have examined the correspondingly designed sEVs for this reason. A crucial molecule in macrophages going through M2 polarization is the signal transducer and activator of transcription 6 (STAT6). Kamerkar et al. created SVs that can be loaded with antisense oligonucleotides targeting STAT6. These SVs were extracted from HEK 293 cells after loading into the cells after design using the engEx platform. The modified sEVs can be absorbed and migrated by cells, and they can successfully inhibit the expression of STAT6 in macrophages, causing them to change from M2 to M1, which has anti-tumor effects (94). Short-course radiation therapy has been used in clinical settings to try and alter the ratio of M1 and M2 phenotypes. Stary et al. compared preoperative tissues with and without radiotherapy and isolated progenitor cells for ex vivo irradiation experiments. They found that short-course neoadjuvant radiotherapy in patients with rectal cancer led to a change in macrophages towards the M1 phenotype and an increase in the M1/M2 ratio. They isolated the EVs from irradiated or unirradiated DLD-1 cells and co-cultured them with M2 macrophages for 48 hours to investigate if the change in the TME by short-course radiation is linked to EV-mediated effects by irradiated cancer cells. They discovered that M2 macrophages tended to change to the M1 phenotype (98). The research suggests that treating colorectal cancer through macrophage transformation to M1 phenotype will be crucial.

### 5.3 Pancreatic cancer-derived extracellular vesicles after artificial modification or treatment

Approximately 80%–90% of pancreatic cancers are pancreatic ductal adenocarcinoma (PDAC), which has a 5-year survival rate of about 8%–25%. PDAC is a highly aggressive malignancy. Increasing its diagnostic sensitivity and treatment strategies is vital because it is typically found in advanced stages or has spread during diagnosis. Su et al. discovered that sEVs generated from pancreatic cancer Panc-1 cell line may change the polarization of macrophages from M1 to M2. This discovery prompted them to co-culture sEVs from Panc-1 cells containing miR-155 and miR-125b-2 with IL-4-induced M2 macrophages. They found higher levels of miR-155 and miR-125b-2 and increased IL-1β/Arg-1 and iNOS/Arg-1 ratios in macrophages. This confirms the shift of the M2 phenotype to M1 phenotype repolarization, which subsequently exerts anti-tumor invasion and metastasis effects (99).

### 5.4 Mesenchymal stem cell-derived extracellular vesicles

Mesenchymal stem cells (MSCs) are pluripotent stem cells extensively used in clinical settings and have the capacity for various differentiation and self-replenishment (100). MSCs come from various tissues, including the umbilical cord, bone marrow, muscle and adipose tissue (101). MSCs are extremely desired transporters with a significant affinity for inflammatory tissues and tumors. By secreting anti-inflammatory substances through autocrine and paracrine secretion, it can readily localize to the injury site and speed up the healing process of wounds (102, 103). MSCs-EVs exhibit significant advantages over typical MSC cell therapy regarding immunogenicity, safety and stability. They have also significantly progressed in treating certain diseases, such as cancer (104), Covid-19 (105), and neurological disorders (106).

The components carried by EVs, which have anti-tumor effects when released to convey anti-tumor mediators, make stem cell EVs therapy a double-edged sword (107). Chen et al. co-cultured HT-29 colorectal cancer cells and macrophages with human umbilical cord MSCs. Their sEV-borne miR-1827 inhibited SUCNR1 expression, preventing macrophage M2 polarization and colorectal cancer cell growth. The combination of MSCs-sEVs-miR-1827 decreased tumor size, reduced M2 polarization markers in macrophages within tumor tissues, and lowered the number of metastatic tumor lymph nodes in the liver of tumor-bearing mice. Concurrently, they conducted related *in vivo* experiments in nude mice. Stem cells generated from human umbilical cord mesenchymal stem cells have anti-tumor and anti-tumor liver metastatic properties (108). Liu et al. co-cultured MSCs-sEVs with macrophages, which inhibited the M2 polarization of macrophages. They then introduced the co-culture supernatant to glioma cell lines, which postponed cell proliferation and invasion. Additionally, they injected MSC-sEVs into mice harboring cancers and observed that the tumors grew lighter. When they dissected the tumor nodules in the lung tissue of the mice, they discovered that the MSC-injected mice had fewer lung tumor nodules overall. MSC-sEVs, therefore, possess anti-tumor and metastatic quality (109). However, stem cell-derived EVs can potentially have pro-tumorigenic effects, primarily associated with the source of the cargo or MSCs they transport. For instance, miR-21-5p in MSC EVs from human bone marrow targets PTEN in macrophages, leading to M2 polarization induction suppressing immune function in lung cancer. To inject MSC-EVs overexpressing miR-21-5p into mice using a nude mouse xenograft model, they increased the formation of tumors (110).

According to the studies above, tumor-derived EVs can also have anti-tumor effects following specific treatments. The human body has a variety of normal cells, including immune cells and stem cells, which are sources of EVs that can treat cancers. These many EV sources can be given to target cells more effectively and have the common benefit of not breaking down enzymatically in the body or causing first-pass metabolic effects (111). Unedited tumor-derived EVs are primarily utilized as therapeutic targets to increase therapeutic efficacy since they primarily control immune cells or transmit drug resistance. Tumor-derived EVs with contents altered through electroporation can prevent immunogenic reactions to cancer by directly reprogramming immune cells to produce anti-tumor effects. There are practical issues with this method of engineering EVs, and the host may reject the cargo-loaded EVs. Naturally, anticancer EVs are secreted by many normal immune cells, which have been altered to create innovative tumor vaccines. Nevertheless, EVs immune cells might be cytotoxic.

## 6 Conclusion

**Figure 2:**
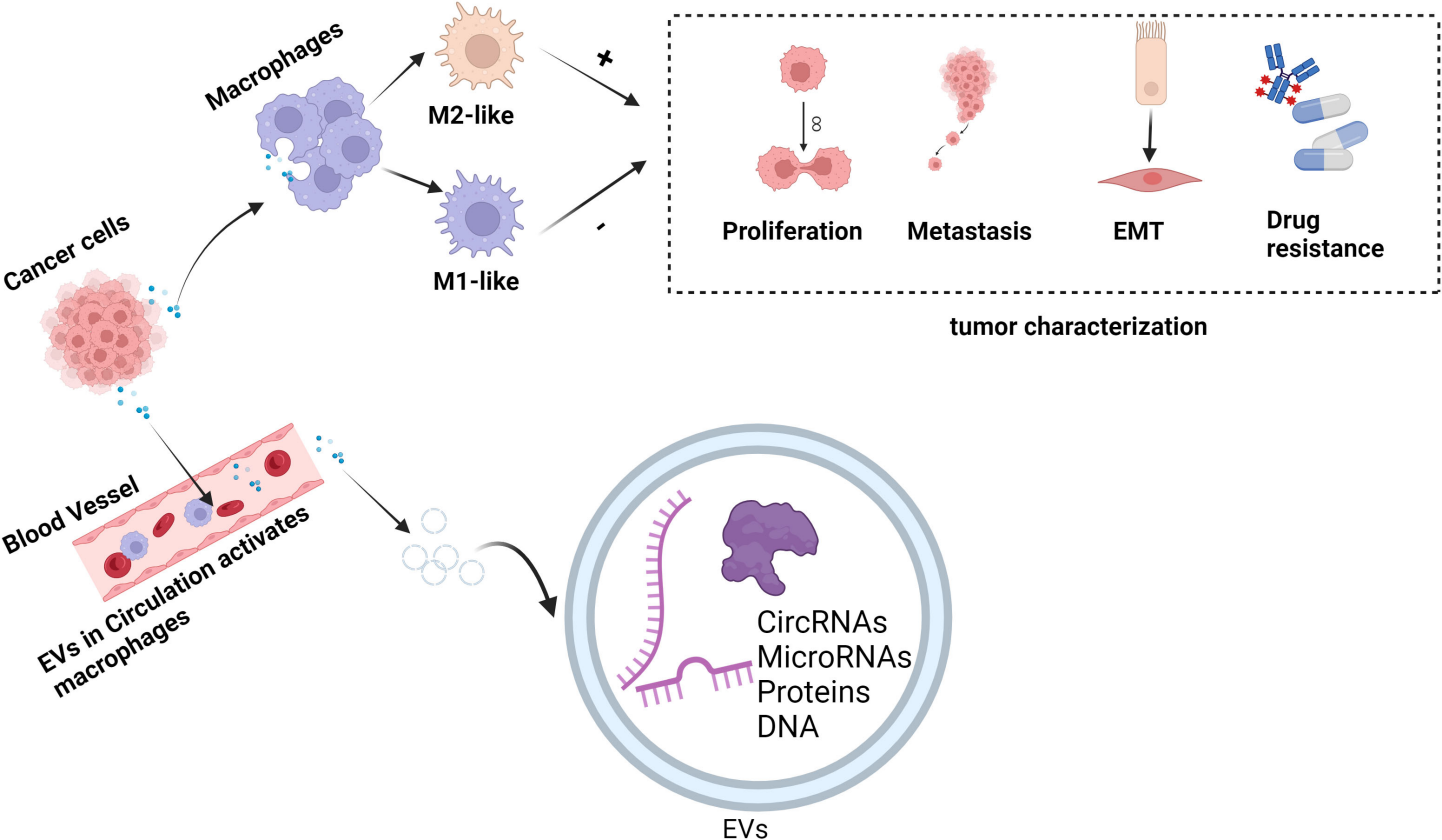
EVs regulate the role of macrophages in tumor pathogenesis. EVs exert anti-tumor or pro-tumor effects by carrying genetic material delivered to macrophages and regulating their polarization to M1 or M2 phenotypes. In the figure, “+” represents promotion and “-” represents inhibition. (This figure was created with Biorender.com.)

In conclusion, EVs show promise in treating and diagnosing several diseases, including oncological diseases. By transporting a range of genetic material, including DNA, RNA, and proteins, and transferring genetic material between donor and recipient cells, EVs can be involved in cellular communication. According to earlier research, EVs are crucial to the pathophysiology of various illnesses. Because EVs are difficult for enzymes to break down in bodily fluids—blood, urine, amniotic fluid, tear fluid, cerebrospinal fluid, etc.—they are found in a wide range of bodily fluids and are crucial for the early diagnosis of tumors. Urine EVs are early diagnostic markers for prostate cancer, while plasma EVs are specific diagnostic markers for lung adenocarcinoma. Tumor phenotype and metastatic potential are assessed by analyzing genetic material composition in tumor EVs from various samples. By targeting macrophages, the EVs released by tumor cells can change their phenotype, impacting the tumor microenvironment and controlling the tumor’s capacity to spread, invade, and metastasize. Thus, focusing on these EVs makes it easier to create innovative tumor-treating drugs. In terms of therapy, tumor-derived EVs can be altered to include cytokines that can activate macrophages to suppress cancer or even eradicate tumor cells, resulting in pro-inflammatory and anti-tumor outcomes that are summed up in **Figure 2**.

Apart from the previously discussed therapeutic approaches, several investigations have been conducted employing engineered EVs. These EVs carry essential anti-tumor drugs, creating a drug delivery system that effectively transports the active ingredients to the appropriate target cells, thus altering the TME for therapeutic effects. Of course, EVs differ from naturally EVs-modified drug carriers in a few ways, the primary ones being their affinity and lower toxicity. Furthermore, efficient anti-tumor vaccines such as dendritic cell-derived EVs have boosted anti-tumor immunity in breast cancer.

Controlling genes or proteins in EVs from tumor-associated cells provides new possibilities for developing anti-tumor solid agents. Macrophages absorb EVs derived from tumor cells and subsequently release growth factors that promote tumor growth. This allows for the artificial modification of EVs to produce cytotoxic chemokines that destroy tumors. Using EVs as a drug delivery system has several benefits, including the ability to bypass being metabolized by the liver, efficiently reach target cells and cross the blood-brain barrier. However, more research is required to increase the affinity, purity, and yield of EVs for this drug delivery method. The main focus of the study is how host immunity is absorbed by and reacts to the drug delivery system’s EVs. Hypersensitivity reactions happen, among other things, when the host develops an immunological response that rejects EVs. Therefore, more research on the routes or mechanisms mediated by EVs remains promising to optimize new tumor vaccines and targeted drugs.

## Author contributions

LW: Software, Writing – original draft. WW: Software, Writing – original draft. DH: Supervision, Writing – review & editing. YL: Resources, Writing – original draft. ZL: Writing – review & editing. TZ: Resources, Supervision, Writing – review & editing. XW: Funding acquisition, Supervision, Writing – original draft, Writing – review & editing.
